# Repetitive Hops Induce Postactivation Potentiation in Triceps Surae as well as an Increase in the Jump Height of Subsequent Maximal Drop Jumps

**DOI:** 10.1371/journal.pone.0077705

**Published:** 2013-10-11

**Authors:** Julian Bergmann, Andreas Kramer, Markus Gruber

**Affiliations:** Sensorimotor Performance Laboratory, Department of Sport Science, University of Konstanz, Konstanz, Germany; The University of Queensland, Australia

## Abstract

Postactivation potentiation (PAP) has been defined as the increase in twitch torque after a conditioning contraction. The present study aimed to investigate the effectiveness of hops as conditioning contractions to induce PAP and increase performance in subsequent maximal drop jumps. In addition, we wanted to test if and how PAP can contribute to increases in drop jump rebound height. Twelve participants performed 10 maximal two-legged hops as conditioning contractions. Twitch peak torques of triceps surae muscles were recorded before and after the conditioning hops. Then, subjects performed drop jumps with and without 10 conditioning hops before each drop jump. Recordings included ground reaction forces, ankle and knee angles and electromyographic activity in five leg muscles. In addition, efferent motoneuronal output during ground contact was estimated with V-wave stimulation. The analyses showed that after the conditioning hops, twitch peak torques of triceps surae muscles were 32% higher compared to baseline values (*P* < 0.01). Drop jumps performed after conditioning hops were significantly higher (12%, *P* < 0.05), but V-waves and EMG activity remained unchanged. The amount of PAP and the change in drop jump rebound height were positively correlated (r^2^ = 0.26, P < 0.05). These results provide evidence for PAP in triceps surae muscles induced by a bout of hops and indicate that PAP can contribute to the observed performance enhancements in subsequent drop jumps. The lack of change in EMG activity and V-wave amplitude suggests that the underlying mechanisms are more likely intramuscular than neural in origin.

## Introduction

It is well established that the contractile history of a muscle can acutely affect its ability to generate force. Increases in muscle twitch contraction force have been demonstrated following brief (3-10 s) high intensity conditioning contractions of the same muscle [15]. After ten seconds of isometric maximum voluntary contraction (MVC), increases in the peak twitch contraction force of 70% have been reported for the knee extensor muscles [1], up to 47% for the plantar flexor muscles [2,3] and of 142% for dorsi flexor muscles [3]. This increase in twitch torque after a conditioning contraction has been termed postactivation potentiation (PAP) [4]. PAP is not restricted to a specific type of conditioning contraction [4-6]. However, typically maximal isometric [1,7,8] or maximal dynamic [9-11] contractions are performed to induce PAP.

It seems reasonable to assume that the impressive increases in muscle twitch forces after high intensity conditioning contractions translate into clear performance enhancements. However, although a variety of studies have investigated the effects of brief maximal or near-maximal contractions on athletic performance, they show little consensus if PAP can be effectively used for enhancing performance of subsequent sports activities. For instance, Gossen and Sale [8] assessed both PAP and maximal unloaded knee extension velocity following isometric MVC, and found decreased knee extension velocity despite the presence of PAP. Other studies have failed to find any effect (neither positive nor negative) of PAP on athletic performance [12,13], while others were able to demonstrate small performance increases associated with PAP [11,14]. Several mechanisms have been proposed that could be responsible for the discrepancy between large amounts of PAP and small or lacking performance gains. The most prominent one is the PAP-fatigue relationship, i.e., the balance between PAP – which seems to be highest directly after the conditioning contraction and decrease afterwards – and fatigue, which has a similar time course, but will affect performance negatively. Further mechanisms include volume, intensity and type of the conditioning contractions, the time between conditioning contractions and the subsequent athletic activity, as well as the type of the subsequent athletic activity (for review see [15]). The matching of the type of conditioning contraction and the type of the subsequent activity has been suggested to be particularly important in order to achieve PAP-induced performance gains in athletic activities [15,16]. In line with this reasoning, several authors have recommended reactive stretch-shortening cycle movements like drop jumps or hops in the preparation for explosive movements [17,18]. However, despite the potential of reactive movements as a stimulus to induce PAP, the literature is lacking in data about the effectiveness of reactive movements as a conditioning contraction. Therefore, in the present study we chose reactive two-legged hops as a conditioning contraction for subsequent drop jumps, thus closely matching the nature of the conditioning contraction and the subsequent action. Drop jumps were chosen because they are often used as representatives for other stretch-shortening cycle movements [17,19].

In addition to uncertainty about the functional relevance of PAP, the underlying mechanisms have yet to be pinpointed. Mainly, two mechanisms on the muscle level have been proposed: the first explanation is that contractile history affects the phosphorylation of regulatory light chains via the myosin light chain kinase. This makes the actin-myosin complex more sensitive to Ca²^+^, resulting in an amplification of myosin cross bridge activity [6,20-22] and hence causing elevated contractile force generation. The second proposed explanation is that high intensity conditioning contractions cause changes in muscle fiber pennation angles that may contribute to optimize force transmission [23]. In addition to providing an explanation for PAP, both of these mechanisms would also be suitable to explain PAP-induced performance increases without changes in neural input. However, based on H-reflex measurements [24,25], it has been suggested that neural changes occur after conditioning contractions that can also contribute to performance enhancement. An increase of synaptic efficiency after conditioning contractions between Ia afferents and ɑ-motoneurons can result in recruitment of higher order motor units [26,27] and thus an increased efferent output of spinal ɑ-motoneurons. If this is indeed a mechanism that contributes to performance enhancement, it should be particularly visible in drop jumps, as Ia afferent input has been shown to contribute considerably to the electromyographic activity during certain phases of drop jumps – especially during the phase of the short latency response (SLR) – and thus increase leg stiffness [28].

An assessment tool that could help to elucidate neural contributions to PAP-associated performance enhancement is provided with recording of the V-wave. V-waves can be evoked through peripheral nerve stimulation during voluntary muscle contractions [29,30]. Usually, V-waves are normalized to the direct maximal motor response (maximal M-wave: Mmax) elicited by the nerve stimulation, and the resulting V/Mmax-ratio may be taken to reflect the magnitude of efferent motoneuronal output during voluntary muscle activation [29,30]. Consequently, the V/Mmax-ratios following a conditioning contraction that induces PAP should be increased if the underlying mechanism of PAP-induced performance enhancement is an increase of synaptic efficiency between Ia afferents and ɑ-motoneurons, resulting in recruitment of higher order motor units and thereby causing an elevated efferent output of spinal alpha motor neurons.

In summary, we hypothesized that maximal hops would induce PAP in triceps surae muscles and positively affect the performance of subsequent drop jumps. Furthermore, we hypothesized that the magnitude of PAP and increases in drop jump performance would be positively correlated. In addition, V/Mmax-recordings were included to reflect alterations in the magnitude of efferent motoneuronal output potentially involved in PAP-associated performance increases.

## Methods

### Subjects

A total of twelve subjects (7 women and 5 men, means ± SD age: 25 ± 3 years, height: 175 ± 9 cm, mass: 71 ± 11 kg) participated in the present study. The participants had neither lower extremity injuries nor any neurological disorders during the last year. The subjects were recreationally active (5 ± 2 h of moderate intensity physical activity per week), but none had prior experience in performing drop jumps. All participants gave their written informed consent to the experimental procedure. The study was conducted in accordance with the Declaration of Helsinki and approved by the local ethics committee of the University of Potsdam.

### Experimental design

Seven days before the measurements took place, the subjects had to perform a familiarization protocol that consisted of 5 x 10 two-legged hops and 10 drop jumps from a height of 26 cm. Subjects were told to jump barefoot, hands akimbo, without heel contact and to bend their knees as little as possible. In addition, they were instructed to conduct all hops and all drop jumps with short ground contact times and maximal rebound jump height. 

On the measurement day, the test sequence was as follows: after a warm-up (5 min cycling on an ergometer with 80 W), the torque of ten electrically evoked twitch contractions of the triceps surae muscles were recorded with an isokinetic dynamometer. Afterwards, participants had to perform ten maximal hops. Thirty seconds after finishing these hops – the time it took to position the participant in the isokinetic dynamometer – another set of ten twitch contractions was evoked and recorded.

**Figure 1:**
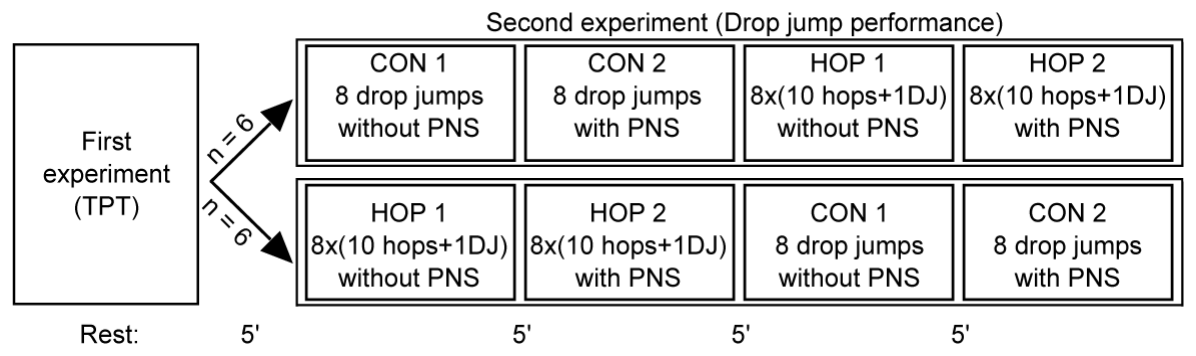
Experimental design. Experimental design of the second experiment: half of the participants first performed 16 drop jumps (DJ) without prior conditioning hops (CON) and then 16 DJs with 10 conditioning hops before each DJ (HOP), whereas the other half performed the two blocks of 16 DJs in the reverse order. Trials with peripheral nerve stimulation (PNS) evoking V-waves were always conducted after the trials without PNS. The time to complete each block of eight DJs was always seven minutes, as the time between two DJs was always one minute, with or without prior hops.

For the second test, subjects performed 16 drop jumps without (CON condition) and 16 drop jumps with a prior set of 10 maximal hops before each drop jump (HOP condition). The time interval between drop jumps was 60 s, with a 5 min rest after each series of 8 drop jumps to reduce fatigue. For the HOP trials, the rest interval between the hops and the subsequent drop jump was 30 s. The order in which the subjects completed the two conditions was randomized and counterbalanced (Figure 1).

In each of the two conditions, the first set of eight drop jumps was performed without any electrical stimuli and served to assess the effect of the conditioning hops on drop jump performance. The second set of eight drop jumps in each condition was performed with peripheral nerve stimulation and served to assess neural contributions to possible performance enhancements. Sets of eight drop jumps were used (instead of just one jump) to increase the reliability of the results.

### Electromyographic recordings

For the electromyographic (EMG) recordings, the skin was shaved, abrased with sandpaper and then cleaned with alcohol. Self-adhesive electrodes (Ambu Blue Sensor P-00-S, Denmark, 34 mm center-to-center distance) were placed over the muscle bellies of soleus (SOL), gastrocnemius lateralis (GAS), tibialis anterior (TA), vastus medialis (VM) and biceps femoris (BF) of the right leg. Five minutes after the placement of the electrodes, the skin impedance between electrodes was checked. Impedance was always below 5 kΩ. The reference electrode was placed over the tibia at the proximal third between the knee and ankle joints. EMG signals were amplified 200x in SOL and GAS and 1000x in TA, VM and BF. The sampling rate of the EMG recordings was 5 kHz. Averaged integrated EMG (iEMG) data were analyzed for the whole ground contact phase of the jump as well as in three specific time intervals: the preactivity phase (PRE) was defined as the time interval from 150 ms prior to touchdown until touchdown. The phase of the short latency response (SLR) was defined as the time interval from 30 - 60 ms after touchdown and the phase of the long latency response (LLR) as the time interval from 90 - 120 ms after touchdown. The time intervals were defined according to previously reported latencies and durations for potential afferent contributions to the EMG [31-33]. To estimate the coactivation of the recorded shank and thigh muscles, the iEMG of TA was divided by the sum of the iEMG of SOL and GAS for each of the phases separately, and the iEMG of BF was divided by the iEMG of VM.

### Peripheral nerve stimulation and V-waves

The two electrodes for the stimulation of the tibial nerve were attached to the right leg: the anode was placed above the patella and the cathode position was determined by moving a positioning electrode in the popliteal fossa until an evoked potential could be visually detected in the SOL raw EMG with the lowest possible electric current. After localization of the spot, the stimulation electrode was attached and additionally fixed with tape. Rectangular voltage pulses of 200 µs duration were delivered from a constant current stimulator (Digitimer DS7AH, Digitimer Ltd., Hertfordshire, UK). Stimuli with increasing electric current were applied to create an H-reflex recruitment curve during normal stance until the maximal M-wave in SOL was reached. From these recordings, the individual latencies of the M-wave and the H-reflex were determined. As V-waves have almost the same latency as the H-reflex [30], the H-reflex latency was used to time the V-waves. For electrical stimulation of M-waves and V-waves during drop jumps and to elicit twitch torques in the isokinetic dynamometer, stimulus current strength was set to 150% of Mmax to ensure the recruitment of all motoneurons [34].

A photoelectric light barrier system (Optojump, Microgate, Bolzano, Italy) was used to trigger the electric stimulations during the drop jumps. Two of these eight stimulations per condition were timed in a way that the V-wave coincided with the individual peak of the SLR, and two were timed so that the V-wave coincided with the peak of the LLR. Two of the remaining four stimulations were timed so that the M-wave – used for normalizing purposes – coincided with the peak of the SLR, and the last two were timed so that the M-wave coincided with the peak of the LLR.

### Muscle twitches recorded in the isokinetic dynamometer

Resting twitch torques in the right ankle were measured with an isokinetic dynamometer (Isomed 2000®, Ferstl GmbH, Hemau, Germany) in a supine position. Hip and knee were fully extended and the foot was positioned on a vertical footplate. The foot, the thigh and the shank were strapped tightly with belts and the shoulders were attached with pads to the device. Triceps surae muscle twitches were evoked by single, rectangular stimuli with a pulse duration of 200µs. The stimulation intensity was 150% of the stimulation current strength necessary to evoke Mmax. The twitch peak torques (TPT) were normalized to the mean of the 10 TPT recorded before the conditioning hops (baseline). Similarly, the peak-to-peak amplitudes of the corresponding M-waves were normalized to the mean of the 10 baseline M-waves recorded before the conditioning hops. The twitches were evoked with an inter-stimulus interval of 30 s [1].

### Kinematics

During the drop jumps, ankle and knee angles were monitored by custom-made goniometers (Scherle, Freiburg, Germany) that were fixed with tape to the ankle and knee of the right leg. Goniometer data were sampled at 5 kHz. The maximal joint excursions in the ankle and knee joints from touchdown until takeoff were calculated to monitor changes in jumping technique [33]. In addition, root mean square (RMS) values during the phases of SLR and LLR were calculated to monitor potential changes in average joint angles during the phases where M- and V-waves were recorded.

### Vertical ground reaction forces (GRF)

Drop jumps were performed on a force plate with a split platform (Leonardo Mechanography GRFP, Novotec Medical, Pforzheim, Germany). Force signals were generated with an output frequency of 800 Hz and were upscaled to 5000 Hz. The force signal of the right platform was used as a trigger signal for EMG and kinematic data, which were all recorded synchronously via a Labview-based data acquisition software (Imago, PfiSoft, Freiburg, Germany). Contact times and rebound flight times were calculated with the Leonardo Mechanography Research Edition software. The performance index was calculated as follows: performance index = rebound flight time / contact time. Rebound jump height was determined using the flight-time method (jump height = 1/8 x g x t^2^, where *g* is Earth's standard acceleration due to gravity and *t* is the air time) [35,36]. 

### Statistical analyses

Kolmogorov-Smirnov tests were performed for each of the analyzed variables, revealing no significant deviances from the normal distribution. Consequently, one-factor (time, 10 levels) repeated-measures analyses of variance (ANOVA) were performed to analyze whether the amplitudes of the TPT or the associated M-waves differed over time. In case of significant main effects, Bonferroni corrected post hoc tests were calculated to determine significant differences between each of the ten post values and the last value before the conditioning hops. If Mauchly’s test of sphericity yielded significant results, Greenhouse-Geisser corrections were applied. Differences between CON (drop jumps without prior hops) and HOP (drop jumps with prior hops) regarding EMG, kinematic and GRF parameters were analyzed with paired samples student’s t-tests. When comparing HOP and CON, the mean values of 8 jumps from each condition were used for evaluation. Pearson analysis was used to correlate changes in TPT and drop jump rebound height. Coefficients of variance (CV) were included in the manuscript whenever test reliability was considered to be important. CVs were calculated as follows: the standard deviation of one subject’s trials was divided by the mean of the subject’s trials. Afterwards, these CVs were averaged across subjects and multiplied by 100 in order to represent the percent variance of the values. Statistical significance level was set at *P* < 0.05. For all the statistical analyses SPSS 19 (SPSS Inc., Chicago, USA) was used. Group data are presented as means ± standard deviations (SD).

## Results

### Twitch peak torques

**Figure 2:**
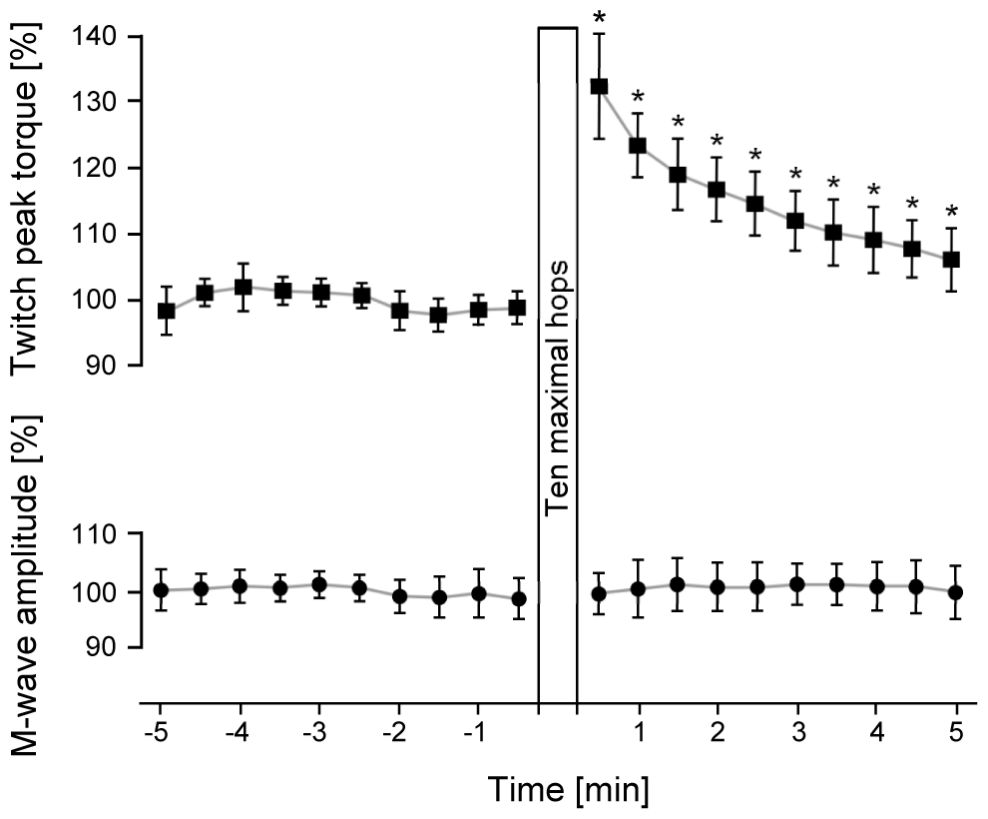
Twitch peak torques and the corresponding M-waves before and after the conditioning hops. Upper part: Resting mean twitch peak torques (TPT) of all participants before and after the ten maximal hops that served as conditioning contraction. All values are normalized to the mean of the 10 TPT prior to the conditioning hops. As hypothesized, there was only a significant main effect of time after the hops. A * symbol indicates a significant difference in the post-hoc analysis. Lower part: Resting mean peak-to-peak amplitudes of the corresponding M-waves (resting Mmax) normalized to the mean of the ten M-waves obtained prior to the conditioning hops. No significant main effect of time was shown.

The mean TPT before the conditioning hops showed stable results by values ranging between 98 - 102% (CV=3%). Following hopping, TPT were enhanced compared to pre values and there was a significant main effect of time for the ten TPT after the conditioning hops (F(_9,99_)=35.7, p<0.001, η^2^=0.76). The grand mean of the TPT in CON condition was 22.1 ± 4.9 Nm. The mean increase in the first recorded TPT 30 s after the conditioning hops was 32 ± 8% (29.3 ± 7.2 Nm) and decreased continuously to 24 ± 5% (27.3 ± 6.0 Nm) after 60 s, 19 ± 5% (26.3 ± 5.9 Nm) after 90 s, 17 ± 5% (25.7 ± 5.3 Nm) after 120 s, 15 ± 5% (25.2 ± 5.1 Nm) after 150 s, 12 ± 5% (24.6 ± 5.0 Nm) after 180 s, 10 ± 5% (24.0 ± 4.9 Nm) after 210 s, 9 ± 5% (23.7 ± 4.8 Nm) after 240 s and 8 ± 4% after 270 s. Five minutes after hopping, peak twitch torques were still significantly 6 ± 5% (23.4 ± 4.8 Nm) higher compared to baseline. All TPTs after the conditioning hops were statistically different from the reference resting TPT prior to the conditioning hops (p<0.05, see Figure 2). Peak-to-peak amplitudes of the corresponding M-waves showed very stable results: there was no significant main effect of time, neither before (F(_9,99_)=0.72, p=0.47, η^2^=0.06, CV=3%), nor after the conditioning hops (F(_9,99_)=0.53, p=0.67, η^2^=0.05, CV=4%). The grand mean of the Mwaves in CON condition was 12.25 ± 0.08 mV and in HOP condition 12.40 ± 0.05 mV.

### Jumping performance

Rebound jump height was 12% higher (2.7 cm; *p*<0.05) in HOP condition compared to CON. In CON condition, the mean rebound jump height was 22.6 cm ± 0.4 cm (CV=5%) compared to 25.3 cm ± 0.5 cm (CV=6%) when the drop jumps were performed directly after the conditioning hops (HOP). Contact times did not differ significantly between conditions (*p*=0.38; 198 ms ± 24 ms; CV=6% in CON and 203 ms ± 26 ms, CV=6% in HOP). The increase in performance index for HOP compared to CON (+4%) did not reach the significance level (p=0.25). Moreover, mean GRF values showed no significant differences between CON and HOP (p=0.95). An overview of the performance outcome variables between the conditions is given in Table 1. The changes in these performance parameters from CON to HOP were not significantly affected by gender. 

**Table 1:** Performance characteristics of the drop jumps performed without (CON) or with prior hops (HOP).

	**CON**	**HOP**	**difference**
**contact time** [ms]	198 ± 24	203 ± 26	2 ± 8%
**rebound flight time** [ms]	427 ± 41	452 ± 48	6 ± 6% *
**Performance Index**	2.18 ± 0.28	2.27 ± 0.38	4 ± 10%
**rebound jump height** [cm]	22.6 ± 0.4	25.3 ± 0.5	12 ± 11% *
**max. vertical GRF** [N]	3781 ± 811	3786 ± 857	0 ± 9%

Grand mean (± SD) of the performance characteristics of the drop jumps without PNS with (HOP) and without (CON) prior conditioning hops, as well as the difference between these two conditions. A * symbol indicates a significant difference.

**Figure 3:**
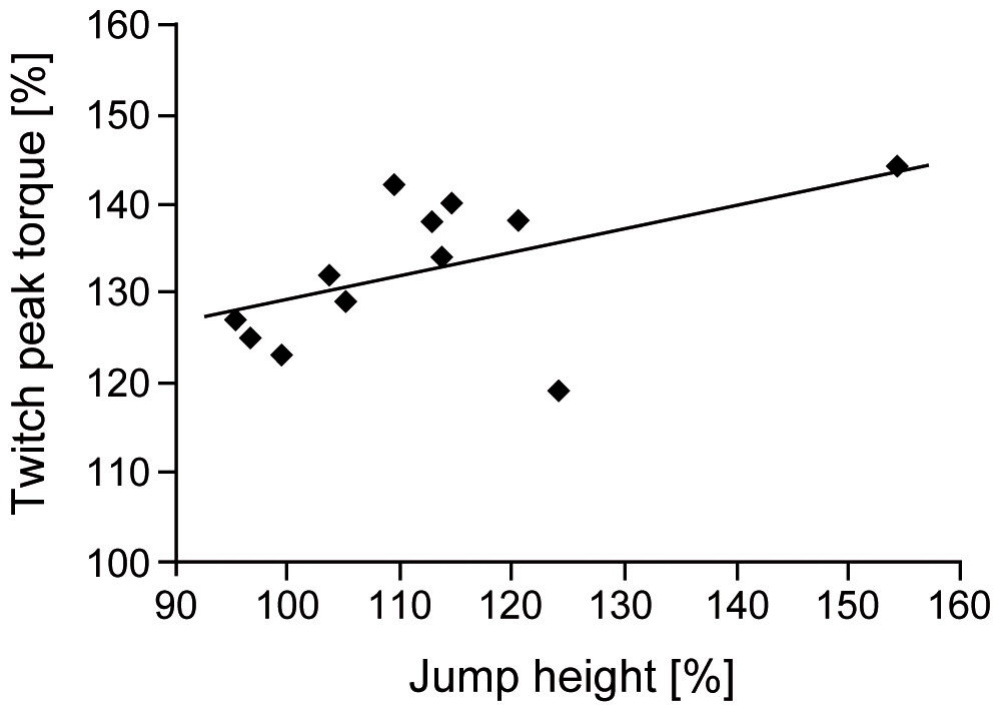
Relationship between changes in twitch peak torque and changes in drop jump rebound height. Relationship between individual changes in twitch peak torque (measure of PAP) and changes in drop jump rebound height for all 12 participants. The increase in twitch peak torque refers to the difference between baseline values assessed before conditioning hops and the first twitch peak torque measured 30 s after the conditioning hops. The change in drop jump rebound height was calculated as the average individual difference between control drop jumps without prior conditioning hops and drop jumps with prior conditioning hops. Note that removing the two outliers would only strengthen the correlation (r^2^ = 0.69 instead of r^2^ = 0.26).

Normalized TPT (reflecting the amount of PAP) and individual changes in drop jump rebound height were positively correlated (r^2^=0.26, p<0.05; r^2^=0.69 when removing the two outliers) (see Figure 3).

### Neuromuscular activity

**Figure 4:**
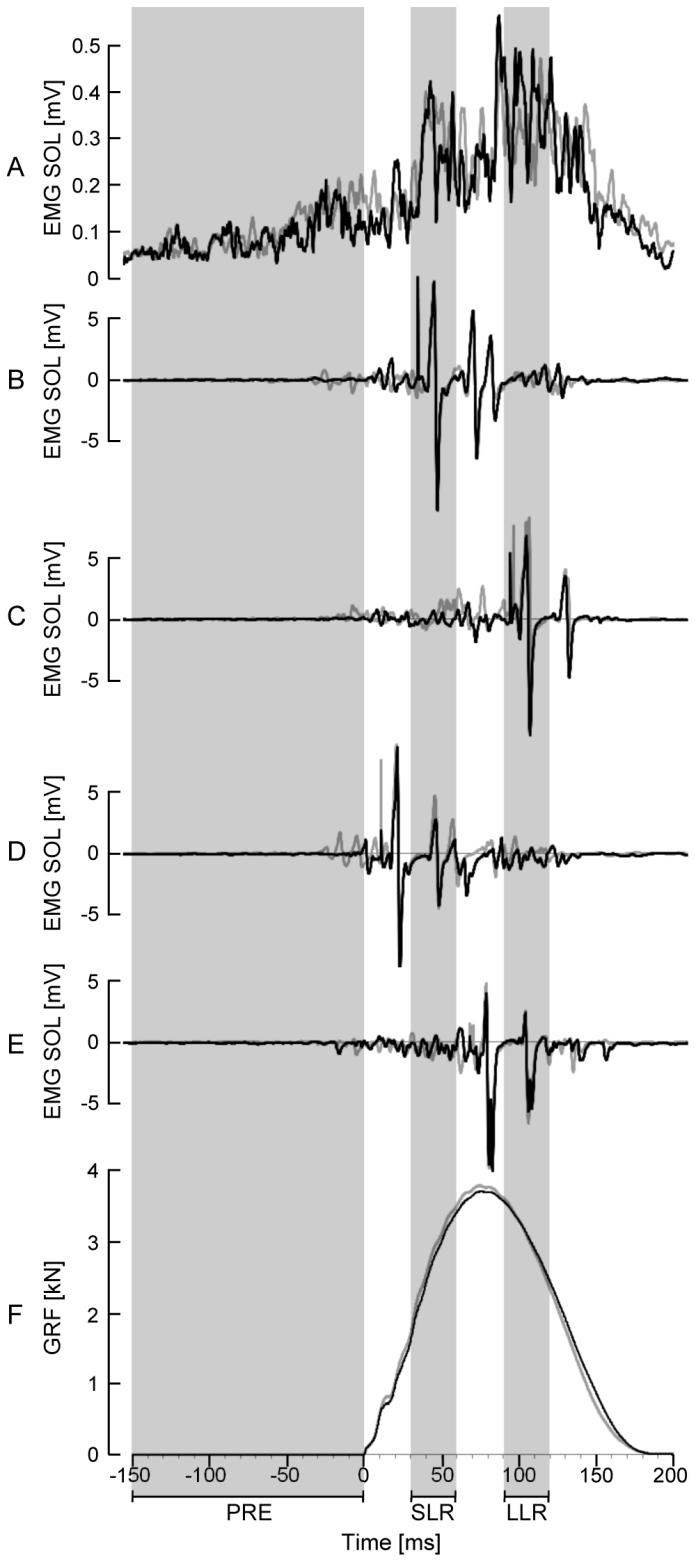
Representative EMG, V-wave and vertical ground reaction force recordings. Averaged rectified EMG (A), evoked potentials (B-E) and GRF (F) of the drop jumps of a representative subject. Black curves represent drop jumps without prior conditioning hops (CON) and the grey lines represent drop jumps after conditioning hops (HOP). The evoked potential (B-E) were recorded as follows: M-wave timed to coincide with the peak of the SLR (B) or the LLR (C), and V-wave timed to coincide with the peak of the SLR (D) or the LLR (E).

No statistical differences between CON and HOP were found for the iEMG values of any of the recorded muscles, neither for the total ground contact phase of the jumps, nor during PRE, SLR and LLR, nor when calculating the coactivation (see table 2). The analysis of the V/Mmax-ratios during SLR and LLR did not reveal statistically significant differences between HOP and CON either (p=0.44 during SLR and p=0.64 during LLR). Representative EMG and V-wave recordings are shown in Figure 4.

**Table 2:** Neuromuscular activity during drop jumps performed without (CON) or with prior hops (HOP).

	**total**		**PRE**		**SLR**		**LLR**	
	**CON**	**HOP**	**CON**	**HOP**	**CON**	**HOP**	**CON**	**HOP**
**SOL** [µV*s]	73 ± 14	73 ± 13	11 ± 5	9 ± 4	12 ± 7	14 ± 6	14 ± 4	14 ± 5
**GAS** [µV*s]	61 ± 10	62 ± 15	22 ± 12	24 ± 11	10 ± 6	10 ± 4	11 ± 3	11 ± 2
**TA** [µV*s]	21 ± 9	22 ± 7	13 ± 6	15 ± 7	3 ± 1	3 ± 1	3 ± 1	3 ± 2
**VM** [µV*s]	76 ± 39	77 ± 43	7 ± 4	8 ± 3	14 ± 10	16 ± 10	13 ± 7	14 ± 9
**BF** [µV*s]	29 ± 11	30 ± 11	6 ± 3	7 ± 2	3 ± 2	3 ± 2	5 ± 2	6 ± 3
**coactivation shank**	0.16 ± 0.07	0.16 ± 0.07	0.42 ± 0.21	0.45 ± 0.18	0.18 ± 0.12	0.18 ± 0.11	0.13 ± 0.06	0.14 ± 0.06
**coactivation thigh**	0.47 ± 0.25	0.48 ± 0.26	1.15 ± 0.72	1.12 ± 0.67	0.26 ± 0.13	0.29 ± 0.22	0.48 ± 0.20	0.56 ± 0.33
**V/Mmax**	-	-	-	-	0.27 ± 0.25	0.30 ± 0.20	0.53 ± 0.21	0.55 ± 0.18

Grand mean (± SD) of overall iEMG activity in SOL, GAS, TA, VM, and BF as well as the coactivation of the recorded muscles (shank: TA/(SOL+GAS), thigh: BF/VM) and the V/Mmax-ratios in SOL during SLR and LLR. There were no statistically significant differences between HOP and CON.

### Joint kinematics

For CON, the maximum excursion of ankle and knee angle was on average 27 ± 8° and 27 ± 8°, respectively. In HOP, mean maximum excursions of ankle and knee angles were 27 ± 8° and 25 ± 7. No changes were found between HOP and CON for RMS ankle and knee angles during SLR (p=0.41 and p=0.07, respectively) and LLR (p=0.79 and p=0.23, respectively).

## Discussion

In the present study we were able to demonstrate considerable PAP in triceps surae muscles after a bout of ten maximal hops. The same type of conditioning contractions – albeit with a different total volume – caused a 12% increase in rebound jump height of subsequent maximal drop jumps. Changes in PAP and changes in drop jump rebound height were positively albeit weakly correlated, indicating that performance enhancement in drop jumps was partly related to PAP in triceps surae.

When studying PAP, it is important to ensure that the conditions under which PAP or PAP-induced performance gains are assessed, are comparable to the control conditions. The results indicate that this was the case in the present study: the peak-to-peak amplitudes of the M-waves did not differ significantly between trials or conditions, indicating a high reliability and validity of the TPT recordings. This was also true for the M-waves evoked during the drop jumps. Furthermore, the recorded joint angles during the phases of interest (SLR, LLR and total) showed no significant differences across conditions (i.e., HOP vs. CON), which is a requirement for a valid comparison of the EMG during those phases.

The magnitude of PAP in triceps surae 30 s after the conditioning hops – an increase of 32% in TPT compared to baseline recordings before the conditioning hops – is comparable to previously reported PAP effects. Shima and coworkers [2] observed an increase of 31% in TPT of the plantar flexors 30 s after the maximal isometric conditioning contraction and Hamada and coworkers [1] observed an increase of 44% in TPT of the knee extensors 30 s after an isometric maximal conditioning contraction. The slightly higher increase in PAP observed in the study by Hamada and coworkers [1] compared to the studies of Shima and coworkers [2] and the present study can most likely be explained by the different muscle groups examined (knee extensors vs. plantar flexors) and potential differences in muscle fibre composition; as a higher proportion of type II muscle fibres has been demonstrated to increase the amount of PAP [37]. The time course of PAP observed in the two abovementioned studies was also similar to the one observed in the present study, i.e., a roughly exponential decay. However, in both of these studies TPT was additionally recorded immediately after the conditioning contraction. Shima and coworkers [2] reported an increase of 47% two seconds after the conditioning contraction and Hamada and coworkers [1] an increase of 71% five seconds after the conditioning contraction, which was much higher than 30 s after the conditioning contraction. These immediate TPT recordings were possible due to the nature of the conditioning contraction, whereas in the present study the earliest measurement could be taken only 30 s after the conditioning hops because it took that long to reposition the participant in the isokinetic dynamometer. However, given the similarities between the amount of PAP and its time course, it is reasonable to assume that the magnitude of PAP after reactive hops would also be highest immediately after cessation of this conditioning protocol.

Although the first experiment nicely demonstrated the effectiveness of reactive hops as a conditioning stimulus for inducing PAP, this effect has to translate to functional performance increases to be useful from an applied perspective. The second experiment showed that this was indeed the case: the rebound jump height of maximal drop jumps performed 30 s after the maximal reactive hops significantly increased by 12%. In addition, the results of the correlation analysis indicate that this significant performance increase is at least partly due to PAP in triceps surae muscles induced by the conditioning contractions (although one has to keep in mind that the study’s sample size is quite low, which somewhat limits the explanatory power of the correlation analysis; also, TPT was recorded after one bout of 10 conditioning hops, whereas for the drop jumps, there was one bout of 10 conditioning hops per drop jump, i.e., a higher total number of conditioning hops). A PAP-induced performance increase of 12% is quite high when compared to the results of other studies, which frequently show only small performance increases – or even none at all – despite substantial increases in TPT. For example, Jones & Lees [38] used squats at 85% of one-repetition-maximum as a conditioning contraction, but found no significant effects on drop jump performance. French and coworkers [7] demonstrated significant improvements in rebound jump height and vertical peak GRF (by about 5%) in drop jumps following 3 x 3 s isometric MVC of the knee extensors, but no significant performance improvements when the conditioning contraction consisted of 3 x 5 s MVC. Small positive effects (+4%, p<0.05) of a 3 x 5 s isometric legpress MVC as conditioning contractions on rebound jump height in drop jumps were also reported by Güllich & Schmidtbleicher [39]. The fact that the performance increase observed in the present study is at least twice as high as those of other studies using drop jump rebound height as a measure for functional PAP-induced performance gains, supports our hypothesis that matching the nature of the conditioning contraction and the subsequent action enhances functional PAP effects. However, it cannot be excluded that part of the differences are due to other factors, such as the rest period between conditioning contractions and the execution of the drop jump or the instructions given about jumping technique. Another point is that in hops more than one muscle group might benefit from PAP effects. PAP was only measured in triceps surae but may possibly also be present in the knee extensor muscles and other muscles that contribute to jump performance. It is also important to note that our performance measure, the jump height of the eight drop jumps, was calculated as the mean of the eight DJs. Therefore, it cannot be excluded that the sets of ten hops and one DJ had effects on the next DJ, either positive due to PAP or negative due to fatigue. However, these possible effects were probably small, as neither the jump height nor the peak GRF over the eight jumps showed a significant effect of time.

Despite the impressive increase in drop jump rebound height after the conditioning hops, the increase in the performance index of the drop jumps, i.e., the ratio of rebound flight time and ground contact time, failed to reach the level of significance. For stretch-shortening cycle activities without narrow time constraints, this is not an issue. However, if there is only a very limited amount of (ground contact) time available to transmit the forces generated by the muscles – for example during a sprint – a high performance index has been suggested to be important [35,36]. One likely reason for the differences regarding the performance increases between rebound jump height (+12%, significant) and performance index (+4%, not significant) is that rebound jump height increases with the square of rebound flight time, whereas the performance index only increases linearly. Combined with the fact that in the present study the ground contact time of the drop jumps was slightly prolonged (though not significantly), this probably explains the major part of the observed differences.

Furthermore, it is possible that the rebound jump height of the drop jumps would have increased even more if they had been performed directly after the conditioning hops instead of 30 s after the hops. This assumption is based on the observed time course of PAP (highest effects during the first recording 30 s after the conditioning hops, followed by an exponential decay) and the positive correlation between the amount of PAP and changes in drop jump rebound height [15].

It seems reasonable to assume that the effect of conditioning contractions on the subsequent performance can depend on the training level of an individual. The participants in the present study were only recreationally active and therefore the results cannot be directly conferred to athletes. However, there is evidence that strength- or power-trained athletes benefit even more from prior conditioning contractions than participants that are not especially trained in this domain (for a review see [15]). It has been suggested that this relationship between strength and power training level and the performance increases after conditioning contractions can be explained by a higher proportion of type II fibers in strength- and power-trained athletes, which lead to higher twitch potentiation [37]. Consequently, it seems possible that power and strength trained athletes show even higher increases in drop jump performance after a bout of conditioning hops than the recreationally active subjects participating in the present study. 

In addition to assessing the effectiveness of reactive hops as a conditioning contraction for PAP and PAP-associated performance increases, the aim of the present study was to elucidate the mechanisms responsible for PAP-associated performance gains. More precisely, we wanted to investigate whether an increase of synaptic efficiency between Ia afferents and ɑ-motoneurons, resulting in recruitment of higher order motor units [27] can be considered to be a likely mechanism explaining PAP-associated performance gains.

We were not able to detect higher EMG activities or reduced coactivations, neither in the different muscles nor in the different contraction phases. However, this does not necessarily exclude neural modulations after the conditioning hops. It has been shown that the relationship between EMG amplitude and muscle force is not always linear. Especially for high force levels, enhanced motoneuron recruitment and higher discharge rates of already recruited motoneurons do not necessarily translate to increased EMG activity [40]. It has been shown that compared to the EMG activity during isometric MVC, EMG activity during maximal drop jumps amounts to 70-80% during the SLR phase and around 100% during the LLR phase [41]. Consequently, the EMG activity during drop jumps does not necessarily reflect changes in motor unit recruitment or discharge rates. For this reason, we recorded electrically evoked V-waves to test for changes in motoneuron recruitment and firing properties not apparent in the normal EMG. The V/Mmax-ratio reflects the overall magnitude of efferent motor output due to activation of motoneurons via central descending drive [29,30]. Hence, an increased Ia synaptic efficiency and/or additional motoneuron recruitment would result in an increased V/Mmax-ratio. In addition, V-waves can be timed precisely during the distinct EMG bursts that occur during the ground contact in the drop jumps. The exact timing is of interest because it has been demonstrated that the Ia afferent transmission is modulated throughout the ground contact. For the soleus muscle, studies using peripheral nerve stimulation demonstrated higher H-reflexes at touchdown that decreased towards the push-off phase [41,42]. It has been argued recently that this progressive decline of the H-reflex amplitude could be an indication that the activity of the triceps surae muscles depends more on Ia afferent transmission during the early phase of ground contact compared to the later phases [43]. With the V-wave it was possible to test for neural modulations at high contraction levels and during two distinct contraction phases where we expected differences in the magnitude of efferent motoneuronal output.

Consequently, in this study, we elicited V-waves in SOL coinciding with the peaks of the SLR and LLR during maximal drop jumps. The V/Mmax-ratios of the V-waves elicited in the present study during the LLR (0.5 ± 0.2, CV = 20%) were comparable to those reported in previous studies for maximal isometric contractions [30,34]. V/Mmax-ratios elicited during the SLR were lower and more inconsistent (0.3 ± 0.2, CV = 21%). Assuming that the V/Mmax-ratio reflects the overall traffic of action potentials traveling along the axons of alpha-motoneurons at the time of the measurement, the higher V/Mmax-ratio during the LLR compared to the SLR might indicate higher recruitment and/or higher firing frequencies of active motoneurons during the LLR. An alternative explanation for the lower V/Mmax-ratios during the SLR compared to the LLR could be that a higher proportion of Ia afferents are refractory during this phase. It has been suggested that the stretching of muscle spindles, which occurs at touchdown, elicits almost synchronous activity in the Ia afferent pathway leading to a burst like activity (SLR) in the homonymous muscles SOL and GAS [44]. In consequence, the probability that an Ia afferent fiber is refractory should be higher when the stimulation is timed to coincide with the SLR compared to a stimulation that is timed to coincide with the LLR. This could explain why V-waves were much lower during the SLR compared to the LLR whereas the overall EMG activity was similar (see Table 2).

We should acknowledge that there are some methodological restrictions in measuring the V-wave during maximal drop jumps. It was only possible to elicit two V-waves and M-waves due to the limited number of total trials our subjects could perform without getting fatigued. During the SLR, the V-waves were much more variable compared to the V-waves elicited during the LLR. Again, the greater variability might be a result of higher and more synchronous Ia afferent transmission during the SLR. According to timing of touchdown and stimulation this could result in almost total extinction of the V-wave or almost no interference with natural Ia activity, increasing the variability specifically during the SLR.

Since no significant changes could be detected when analyzing the V/Mmax-ratios with and without prior conditioning hops, the results of the present study do not support the assumption that neural changes following the conditioning hops is a likely mechanism responsible for the performance enhancements. Keeping in mind that small changes in the neuromuscular activity are difficult to detect due to the inherent variability of EMG recordings, this is in line with the observations by Jones & Lees [38], who found no significant differences in EMG activity when comparing drop jumps with and without prior conditioning contraction. Therefore, it is more likely that mechanisms at the muscle level are responsible for the present PAP and PAP-induced performance enhancements, such as the phosphorylation of myosin regulatory light chains [6,20], or changes in pennation angle [23]. Other possible explanations include changes in muscle (co-) activation timing or PAP in other muscles not measured in this study, which might have contributed to performance increases as well. 

In summary, we showed that a bout of ten maximal reactive hops caused a substantial PAP effect in the triceps surae muscles, which translated to functional performance increases, manifested as an increase in drop jump rebound height of the jumps with ten prior conditioning hops. The fact that these performance gains were much higher than in studies using isometric conditioning contractions suggests the importance of matching the type of the conditioning contractions and the type of the subsequent activity whose performance is to be increased. Increased motoneuronal output as a likely mechanism responsible for PAP-induced performance gains could not be confirmed by V-wave and EMG recordings. From an applied perspective, the impressive performance gains in maximal drop jumps achieved in the present study suggest that it may be worthwhile to explore the potential of hops as a preparation for other stretch-shortening cycle type of movements (e.g. long jump, high jump or sprinting) and for athletes with a higher training status.
